# Integration of Chronic Care in a Fragmented Healthcare System: Comment on "Integration or Fragmentation of Health Care? Examining Policies and Politics in a Belgian Case Study"

**DOI:** 10.34172/ijhpm.2022.7143

**Published:** 2022-07-26

**Authors:** Miek Smeets, Karolien Baldewijns, Bert Vaes, Hilde Vandenhoudt

**Affiliations:** ^1^Department of Public Health and Primary Care, Katholieke Universiteit Leuven, Leuven, Belgium.; ^2^Mobilab & Care, Thomas More University of Applied Sciences, Geel, Belgium.; ^3^LiCalab, Thomas More University of Applied Sciences, Geel, Belgium.

**Keywords:** Integrated Care, Chronic Care, Learning Healthcare Network, Macro-Level Barriers, Belgium, Health Policy Reform

## Abstract

The authors of "Integration or Fragmentation of Health Care? Examining Policies and Politics in a Belgian Case Study" present a fresh perspective on the inertia of integrated care (IC) implementation. They conclude that the decisive power in Belgium is fragmented and undermines efforts towards IC. As researchers in integrated heart failure (HF) care and active primary healthcare professionals, we comment on the three policy initiatives evaluated by Martens et al from a bottom-up perspective. A Learning Healthcare Network (LHCN) was established September 2019 to overcome fragmentation, the lack of evaluation and capacity loss each time a pilot project ends. This commentary wishes to illustrate that a LHCN can be a powerful meso-level mechanism to engage in alignment work and to overcome macro-level barriers that are often difficult to change and not supportive of IC.

## Towards Integrated Care in a Fragmented Healthcare System

 Martens et al evaluated three Belgian policy initiatives on integrated care (IC) by applying a stakeholders and processual analysis. They described the organization of Belgian IC focusing on the influence of politics on policy implementation. The authors concluded that the decisive power in Belgium is fragmented and undermines efforts towards IC.^1^ De Maeseneer et al and Gray shared their commentaries on this paper with suggestions on how changes at a macro-, meso- and micro-level could support future integration of care in Belgium.^2,3^ In addition, we would like to add our perspective, as researchers in the field of integrated heart failure (HF) care and active primary healthcare professionals. Our itinerary exposes the consequences of policy decisions on the very slow adaptation of innovative practices in healthcare and how a Learning Healthcare Network (LHCN) can be a means to deal with these macro-level barriers. Although Belgium presents a particular situation, any healthcare system worldwide is challenged with the transition from acute to chronic care.^4^ HF care in Belgium is described as a real-life use case, however, HF can be interchanged by any other chronic disease or multimorbidity.

## The Influence of Belgian Integrated Care Policies From a Bottom-up Perspective

 Martens et al described three Belgian IC policies. First, the type 2 diabetes care trajectory, which was at least moderately supported by many stakeholders as a good first attempt of IC, innovative at that time but not the way to move forward.^1^ A generic approach to provide care for multimorbid elderly is indeed needed.^5^ In order to achieve this, we require new forms of institutional structures or leadership.^2,3^ In addition, there is need to develop new roles and competencies.^6-8^ For example, let’s have a closer look at patient education and empowerment. These are important elements of IC but are only structurally embedded in the diabetes and chronic kidney disease care trajectory in Belgium, which leads to inequity by disease.^1,7^ In primary care, the aim is to implement a model in which advanced practice nurses and primary care nurses receive a modular training to deliver patient education for multimorbid, chronically ill patients.^9^ However, to support nurses in taking up this role in the current healthcare financing system, the “law on the execution of tasks” needs to acknowledge this activity and another fee-for-service nomenclature number needs to be created.^8^ A clear example of policy standing in the way of innovation.

 Second, within the National plan ‘integrated care for better health’ 12 regional pilot projects, aiming to implement IC, were set up in 2018. However, the federal government, which was the most resourceful stakeholder, disregarded the pilots and became one of its strongest opposers.^1^ In essence, regions formed local collaborative working groups to tackle challenges associated with the growing number of chronically ill patients. The concept of these projects was indeed innovative and triggered collaborative governance in multiple regions.^6^ Interestingly, even in regions that were not selected, regular meetings to prepare the project proposal led to a common language and shared vision. In some regions the rejected project plans were still implemented but with different funding sources, in many regions it reinforced local collaborative practice. The pilot projects had to focus on more than one disease also termed multimorbidity. Often, they chose to tackle aspects of care that are currently problematic (care for mental illnesses, all chronic care except diabetes and chronic kidney disease, prevention/positive health). Because there is a clear gap between guideline-directed and actual care for HF in Belgium, two regions selected integrated HF care as a topic. Independently from one another, six other multidisciplinary HF pilot projects were set up in different Flemish regions. This illustrates the quote in Martens et al: “Everything is a pilot” but also underlines the growing awareness for IC and the willingness to collaborate at the micro-and meso-level. Despite this bottom-up willingness for change, evaluation capacity and financial and political support was missing for most projects.^1^ However, the current federal minister of health recently renewed his support towards IC by setting the contours of a new plan, and he seems committed to embed IC into the existing health system.^10^

 As a third policy initiative, the primary care reform was discussed. A central part of this reform was the creation of 60 Primary Care Zones. They were set up in 2019 at a local level to support better coordination and intersectoral collaboration as well as improve planning for larger groups of the population. Martens et al noted high support from stakeholders and policy makers.^1^ Indeed, this restructuring proved to be effective in enhancing multidisciplinary and transmural collaboration to tackle the coronavirus disease 2019 (COVID-19) pandemic.^2^ However, it remains a challenge to engage individual health professionals operating within the primary care zones and to gain their full support for collaborative initiatives to make the population healthy. It is therefore important to solicit their perspectives as well, and assess which form of collaboration works best. An example of good practice is community-oriented care where care professionals form local multidisciplinary teams to serve a neighborhood (5000-10 000 inhabitants), as shown by ‘Zorgzaam Leuven,’ one of the IC projects.^11^

## A Learning Healthcare Network as a Meso-Level Mechanism

 To overcome this fragmentation, the lack of evaluation and the capacity loss when projects end, the eight Flemish multidisciplinary HF projects were united and HeartsConnect was established in September 2019, a LHCN on HF.^12^ LHCNs aim to bring together the collective knowledge of professionals, researchers as well as patients, and distribute information and know-how over large groups of people, in order to accelerate implementation.^13^ The activities of this LHCN also correspond with the definition of a quality improvement collaborative, being a group of experts that unite multiple sites to improve quality of care of a specific health topic, evaluate change and organize activities that promote a collaborative process to learn and share ideas, innovations and experiences.^14^ A systematic review on quality improvement collaboratives showed that they have the potential to significantly improve targeted clinical processes and patient outcomes.^14^

 More specifically, 3-monthly symposia were organized with the participating projects on relevant themes, existing tools were mapped and the best ones were shared. Educational modules were developed to empower primary healthcare professionals involved in HF care. Together with cardiologists of the Belgian Working Group on Heart Failure (https://www.heartfailure.be/en/home), a synthesis of the existing care paths and protocols is currently being made to create one uniform Belgian multidisciplinary HF care path. Moreover, a budget has been allocated to update the HF guideline for general practitioners.

 A LHCN is a good example of a meso-level mechanism that engages in alignment work to overcome macro-level barriers that are often difficult to change and not supportive of IC.^3,6^ Looman et al described that one way to deal with macro-level barriers is accepting them and working within the given regulations.^6^ That is how the network currently tries to overcome the existing macro-level barriers. With respect to the non-existence of nurse-led patient education in primary care for HF and other chronic illnesses in Belgium, a training program for primary care nurses in HF education was developed, despite the current lack of reimbursement and recognition of the role. Candidates for this training are nurses operating as practice assistants in general practice, salaried primary care nurses in larger organizations, nurses from long-term care facilities, etc. This can be a first step of capacity building in primary care, awaiting structural support and educational modules for other chronic diseases. Another frequently mentioned barrier is the lack of rigorous evaluation methods. This leads to unclear results which hinder adaptation and upscaling. Ideally, population health management should be a driver for IC, requiring good and standardized local data, effective information management systems and analytical capability for segmenting population groups based on their different needs. In Belgium there is a large potential to collect and analyze healthcare data. The establishment of a learning health information system where readily available data sources are coupled and feedback is given to patients and healthcare providers on an individual and population-level should be the aim.^3,8,15^ Meanwhile, automatically registered data from participating hospitals and general practices will be used to evaluate the impact of the different projects on regional HF hospitalization rates and the detection of HF diagnosis. It is a stepping stone in growing a culture of continuous monitoring to improve quality of care.^3^

 Most elements described as drivers for successful implementation of IC are incorporated in the LHCN: a stepwise approach, the balance between flexibility and protocols, collaborative governance and distributed leadership, building a multidisciplinary team including new roles and competencies.^6^ However, long-term funding and ICT that promotes collaboration is missed. The latter was one of the main barriers reported by every project in the network underlining the need for an ‘interprofessional integrated goal-oriented electronic health record.’^2,6,8^

 To conclude, Martens et al accurately described how Belgian IC policies do not support integration of care. The establishment of a LHCN is one way to unite multidisciplinary stakeholders and become more visible for policy makers in a fragmented political system. It is an example of a meso-level mechanism engaging in alignment work to overcome macro-level barriers or to work around these barriers.

 The LHCN on HF has been established with the support of the Dr. Daniël De Coninck Fund, managed by the King Baudoin Foundation.

## Ethical issues

 Not applicable.

## Competing interests

 Authors declare that they have no competing interests.

## Authors’ contributions

 MS, KB, BV, and HV decided upon the concept and content of this commentary. MS and KB made the first draft. BV and HV made revisions based on their expertise.
